# Supplementary material for: Profiles of anemia in adolescent students with sports club membership in an outpatient clinic setting: a retrospective study
Source: PeerJ. 2022 Feb 25;10:e13004. doi: 10.7717/peerj.13004 (PMC8884060; doi:10.7717/peerj.13004)
Supplement: Supplemental Information 1 — Median [range] or number (percentage) are shown. *Data on 4 males and 17 females were assessed. Abbreviation; MCV, mean corpuscular volume; MCH, mean corpuscular hemoglobin; n.s., not significant. P values were obtained with Fisher’s exact test for categorical variables and Mann-Whitney U test for continuous variables. [file peerj-10-13004-s001.docx]

**Supplemental table 1. Profiles of laboratory data after classifying iron status among anaemic patients.**

| Variables | Male |  |  |  | Female |  |  |
| --- | --- | --- | --- | --- | --- | --- | --- |
|  | Iron deficiency anemia  (*n* =13) | Non-iron deficiency anemia  (*n* =5) | *p* value |  | Iron deficiency anemia  (*n* =42) | Non-iron deficiency anemia  (*n* =11) | *p* value |
| *Laboratory Data* |  |  |  |  |  |  |  |
| Hemoglobin (g/dL) | 11.7 [8.1 - 12.7] | 12.8 [11.7 - 12.9] | 0.007 |  | 10.9 [6.6 - 11.9] | 11.8 [11.0 - 11.9] | 0.001 |
| Hematocrit (%) | 36.1 [27.0 - 39.9] | 38.4 [34.2 - 39.1] | 0.208 |  | 33.8 [25.9 - 38.0] | 35.3 [32.7 - 36.5] | 0.072 |
| MCV (fL) | 84.0 [67.0 - 91.0] | 92.0 [87.0 - 94.0] | 0.003 |  | 83.0 [58.0 - 94.0] | 89.0 [80.0 - 97.0] | 0.003 |
| MCV <80 | 4 (31) | 0 (0) | 0.278 |  | 16 (38) | 0 (0) | 0.023 |
| MCH (pg) | 26.2 [20.0 - 30.0] | 30.9 [28.4 - 31.5] | 0.003 |  | 26.9 [14.9 - 30.7] | 29.1 [26.8 - 32.0] | 0.001 |
| MCH <27 | 7 (54) | 0 (0) | 0.101 |  | 21 (50) | 1 (9) | 0.017 |
| Reticulocyte (‰) | 10 [7 - 25] | 13 [10 - 16] | 0.336 |  | 13 [6 - 33] | 12 [7 - 21] | 0.757 |
| Reticulocyte >20 | 1 (8) | 0 (0) | 1.000 |  | 2 (5) | 1 (9) | 0.510 |
| Vitamin B12 deficiency | 9 (69) | 0 (0) | 0.029 |  | 20 (48) | 5 (46) | 1.000 |
| Folate deficiency | 0 (0) | 0 (0) | - |  | 8 (19) | 2 (18) | 1.000 |
| Elevation of serum creatine kinase level | 13 (100) | 4 (80) | 0.278 |  | 20 (48) | 6 (55) | 0.745 |
| Low haptoglobin | 5 (39) | 1 (20) | 0.615 |  | 17 (41) | 4 (36) | 1.000 |
| Zinc deficiency* | 4 (100) | - | - |  | 9 (69) | 3 (75) | 1.000 |

Median [range] or number (percentage) are shown. *Data on 4 males and 17 females were assessed. Abbreviation; MCV: mean corpuscular volume, MCH: mean corpuscular hemoglobin, n.s.: not significant. P values were obtained with Fisher’s exact test for categorical variables and Mann-Whitney U test for continuous variables.
