# Supplementary material for: Profiles of anemia in adolescent students with sports club membership in an outpatient clinic setting: a retrospective study
Source: PeerJ. 2022 Feb 25;10:e13004. doi: 10.7717/peerj.13004 (PMC8884060; doi:10.7717/peerj.13004)
Supplement: Supplemental Information 2 — The odds ratios [95% confidence interval] are shown. Abbreviation; BMI, body mass index; MCV, mean corpuscular volume; MCH, mean corpuscular hemoglobin. [file peerj-10-13004-s002.docx]

**Supplemental table 2. Univariate analysis to predict anemia.**

| Variables | Male |  |  | Female |  |
| --- | --- | --- | --- | --- | --- |
|  | Odds | *p* value |  | Odds | *p* value |
| *Patient Characteristics* |  |  |  |  |  |
| Age (years) | 1.1 [0.8 – 1.3] | 0.639 |  | 1.0 [0.8 – 1.1] | 0.724 |
| BMI (kg/m^2^) | 0.9 [0.7 – 1.1] | 0.254 |  | 1.0 [0.8 – 1.1] | 0.704 |
| Menstruation |  |  |  |  | 0.693 |
| Regular menstruation |  |  |  | 1 (ref) | – |
| No menstruation |  |  |  | 0.5 [0.1 – 3.9] | 0.471 |
| Irregular menstruation |  |  |  | 1.1 [0.5 – 2.2] | 0.814 |
| Affiliated Club |  | 0.041 |  |  | 0.756 |
| Others | 1 (ref) | – |  | 1 (ref) | – |
| Track & Field/Athletics | 9.2 [1.2 – 73.5] | 0.036 |  | 1.2 [0.5 – 2.8] | 0.637 |
| Basketball/football | 7.4 [0.6 – 86.7] | 0.110 |  | 1.5 [0.7 – 3.0] | 0.278 |
| Soccer | 9.0 [0.9 – 84.2] | 0.054 |  | 1.3 [0.1 – 13.5] | 0.808 |
| Frequency of practice |  | 0.675 |  |  | 0.446 |
| Less than 5 times a week | 1 (ref) |  |  | 1 (ref) |  |
| 5 times a week or more | 1.6 [0.2 – 13.7] | 0.689 |  | 1.6 [0.4 – 6.2] | 0.463 |
|  |  |  |  |  |  |
| *Laboratory Data* |  |  |  |  |  |
| Hypoferritinemia | 11.0 [3.7 – 32.9] | <0.001 |  | 6.4 [3.1 – 13.2] | <0.001 |
| Vitamin B12 deficiency | 0.8 [0.3 – 2.0] | 0.609 |  | 0.9 [0.5 – 1.7] | 0.773 |
| Folate deficiency | 0 | 0.998 |  | 3.9 [1.5 – 9.9] | 0.005 |
| Elevation of serum creatine kinase level | 11.3 [1.5 – 86.6] | 0.020 |  | 2.6 [1.4 – 4.8] | 0.003 |
| Low haptoglobin | 0.5 [0.2 – 1.4] | 0.205 |  | 1.2 [0.6 – 2.2] | 0.612 |
| Zinc deficiency | 0 | 0.998 |  | 2.1 [0.6 – 6.7] | 0.219 |

The odds ratios [95% confidence interval] are shown. Abbreviation; BMI: body mass index, MCV: mean corpuscular volume, MCH: mean corpuscular hemoglobin.
